# A Cross-Sectional Study Investigating the Relationship Between Alexithymia and Suicide, Violence, and Dual Harm in Male Prisoners

**DOI:** 10.3389/fpsyt.2021.670863

**Published:** 2021-04-29

**Authors:** Laura Hemming, Jennifer Shaw, Gillian Haddock, Lesley-Anne Carter, Daniel Pratt

**Affiliations:** ^1^Division of Psychology and Mental Health, School of Health Science, University of Manchester, Manchester, United Kingdom; ^2^Manchester Academic Health Sciences Centre, Manchester, United Kingdom; ^3^Greater Manchester Mental Health National Health Service Foundation Trust, Manchester, United Kingdom; ^4^Division of Population Health, Health Services Research & Primary Care, University of Manchester, Manchester, United Kingdom

**Keywords:** suicide, violence, forensic, clinical psychology, emotion regulation

## Abstract

**Background:** Suicide and violence are common within male prisoners. One suggested risk factor for both behaviors is alexithymia. Alexithymia describes a deficit in identifying and describing feelings and is also related to externally oriented thinking. This study aimed to explore the relationship between alexithymia, suicide, violence and dual harm in male prisoners.

**Methods:** Eighty male prisoners were recruited from three prisons. Participants were asked to complete a battery of questionnaires including measures of alexithymia (TAS-20), suicide ideation (ASIQ), suicide behavior, violence ideation (SIV), violence behavior, depression (BDI-II), hopelessness (BHS), impulsivity (DII) and anger (NAS-PI). Regression analyses and ANOVAS were conducted to assess the association between alexithymia (and its subcomponents) with six outcomes; suicide ideation, suicide behavior, violence ideation, violence behavior, dual harm ideation and dual harm behavior.

**Results:** Alexithymia was a univariate predictor of suicide ideation, though was not a significant predictor when considered in a multivariate model. Alexithymia was a significant multivariate predictor of suicide behavior. Alexithymia was not a significant multivariate predictor of violence ideation or behavior. There were no significant differences in alexithymia or subscales between those with suicide ideation/behavior alone, violence ideation/behavior alone and those with dual harm ideation/behavior.

**Conclusion:** In male prisoners, alexithymia appears an important univariate predictor of suicide and violence, though the current study suggests no significant contribution above other well-known correlates of suicide and violence.

## Introduction

### Suicide

There are several well-established psychological correlates of suicide. For instance, severity of depression symptoms and hopelessness have been found to have strong relationships with suicide ideation (1–3). Anger and impulsivity have also been established as key correlates of suicide behavior (4–7).

A number of theories of suicide recognize that an immediate antecedent to suicidal thoughts and behaviors is the experience of unmanageable distress (8, 9), and those who experience difficulties regulating emotions are more likely to die by suicide (10). Emotion dysregulation is therefore considered to play a key role in the development of suicidal ideation and behaviors. One specific form of emotion dysregulation found to have an association with suicide outcomes is alexithymia. Alexithymia can be defined as the inability to identify or express emotions (11) and is thought to comprise five main components; (i) a difficulty in identifying one's emotions (ii) a difficulty in describing self-feelings verbally (iii) a reduction or incapability to experience emotions (iv) an externally oriented cognitive style (e.g., lack of fantasy and imagination) and v) poor capacity for fantasizing or symbolic thought (12). The most frequently used measure of alexithymia is the Toronto-Alexithymia Scale (TAS-20), which comprises three main factors; a difficulty identifying feelings; a difficulty describing feelings and an externally oriented thinking style (13).

A previous systematic review found a large effect size in a meta-correlation between measures of alexithymia and suicide ideation and a small effect size in the meta-correlation between alexithymia and suicide behavior (14). The review also found a stronger relationship existed between the subcomponents of “difficulty identifying and describing feelings” as opposed to “externally oriented thinking” with both suicide ideation and behavior. Finally, the review concluded that there might be evidence of clinical variables, particularly depression, impacting the relationship between alexithymia and suicide ideation and behavior. Other meta-analyses have found a medium effect size for the relationship between alexithymia and self-harm outcomes, and have again found the subcomponents of difficulty identifying and describing feelings to be strongest correlates (15, 16).

### Violence

There are a number of well-established psychological correlates of violence. For instance, both impulsivity and anger are known to be associated with violent behavior (17–19).

Alexithymia and emotion dysregulation have also been purported to associate with violence to others. For instance, some theories of violence suggest that an inability to regulate anger may lead to violence (20, 21). Specifically, a relationship has been found between alexithymia and violence in a range of populations including; people with substance dependence (22, 23), university students (24, 25), males with antisocial personality disorder (26) and veterans with traumatic symptoms (27). Similar to findings on the relationship between alexithymia and suicide, most studies found a closer association with the difficulty identifying and describing feelings subcomponents of alexithymia with violence.

### Dual Harm

Dual harm is a term used to describe individuals who engage in acts to hurt both themselves and others (28). A wealth of evidence exists to suggest that those who engage in acts of harm to themselves are more likely to have previously engaged in acts of harm to others and vice versa (29–31). Furthermore, researchers have reported that as many as 23 psychosocial risk factors may be causally implicated in both suicide and violence, including for instance hopelessness and impulsivity (32, 33), whilst some researchers have noted that those who engage in dual harm may be both qualitatively and quantitatively different from those who engage in sole harmful behaviors (28). Specifically, there is evidence to suggest that those who engage in dual harm may have greater difficulties managing their emotions, may be more likely to have experienced negative events during childhood and more likely to possess antisocial traits (34–37).

### Prison Populations

It has been reported that suicide rates in male prisoners, both violent and non-violent, are three to six times higher than in the general population (38–40). In 2019, there were 80 self-inflicted deaths and 63,328 self-harm incidents in prisons across England and Wales (41). In the same year there were 32,669 assault incidents, of which 3,813 were serious assaults (41). It has also been reported that there is a high prevalence of dual harm within male prisoner populations, with one study reporting that 60% of prisoners who self-harmed had a history of aggression, and that prisoners who self-harmed were three times more likely to be aggressive than those with no history of self-harm (42). Furthermore, rates of alexithymia amongst prisoners have been estimated to range between 31 and 47% (43–46); almost three times higher than the estimated prevalence of between 10 and 19% in the general population (47–52).

Despite this, to date, the only research which has examined the relationship between alexithymia and suicide and violence in a prison population has been qualitative in nature. For instance, a study of interviews with prison staff found that prisoners struggle to identify, understand and communicate their feelings which could lead to unregulated emotions. These unregulated emotions were found to be resolved using maladaptive coping strategies such as using drugs and alcohol or harming self or others (53). Furthermore, a study with prisoners found that a combination of internal and external factors can lead some prisoners to avoid talking about their feelings in prison. This can lead to a build-up of emotions, which is experienced either as a “void” of emotions or an “emotional overload.” Each of these experiences was reported to be frequently resolved by hurting self or others (54).

### Aims of the Study

This is the first study that aims to quantify the relationship between alexithymia and suicide, violence and dual harm in a male prisoner population. Based on previous literature, the following core hypothesis was proposed:

H1: A higher score on a measure of alexithymia will be associated with a higher score on a measure of suicide ideation

In addition to this, a number of exploratory hypotheses were proposed:

H2: A higher score in alexithymia will be associated with an increased odds of having experienced suicidal behavior in the past 2 weeks

H3: A higher score in alexithymia will be associated with an increased odds of scoring “high” in violence ideation

H4: A higher score in alexithymia will be associated with an increased odds of having experienced violent behavior in the past 2 weeks.

H5: Those who have experienced dual harm ideation will have higher scores in alexithymia than those who have experienced suicidal or violent ideation alone

H6: Those who have experienced dual harm behaviors will have higher scores in alexithymia compared to those who have experienced suicidal or violent behaviors alone.

Additionally, these hypotheses were further tested whilst also controlling for known covariates of suicide and violence, and were examined using the subscale scores of alexithymia along with the alexithymia total score.

## Materials and Methods

### Study Design

A cross-sectional questionnaire design was used. For hypotheses 1–4 the outcome variables were suicide, violence and dual harm ideation and behavior. The predictor variable was alexithymia. Depression, hopelessness, impulsivity and anger were included as confounding variables. For hypothesis 5, alexithymia, depression, hopelessness and anger were the outcome variables and category of ideation was the predictor variable. For hypothesis 6, alexithymia, impulsivity and anger were outcome variables and category of behavior was the predictor variable.

### Patient and Public Involvement

A patient and public involvement group comprising five individuals with lived experience of incarceration was recruited to assist with the design, interpretation of results and dissemination of this study. Specifically, the group was responsible for assisting with creation of participant documents such as the participant information sheet, consent form and study advertisement posters. Further, they were heavily involved in choosing questionnaire measures for the study, by trialing several proposed questionnaires for each construct measured and helping to choose one based on both ease and emotional response to questions. Finally, one group member was involved in assisting with the interpretation of findings and also in reviewing the participant summary detailing the findings.

### Setting

Data were collected from participants in three male prisons in the North West of England. Data collection began in April 2018 and finished in January 2020.

### Participants

Staff in safer custody teams^1^ at each of the three sites identified potential participants. Participants were eligible to take part if they: (i) were aged 18 years or over; (ii) had been residing in a prison in North West of England for at least 1 week; (iii) possessed sufficient English language skills, as assessed by the researcher; (iv) possessed sufficient mental capacity to provide informed consent, as assessed by the researcher. In addition, participants must have fulfilled at least one of the following criteria: (v) identified by a member of prison staff or via self-report as having recently engaged in an actual or expressed suicide attempt or as at risk of engaging in an attempt due to suicide ideation; (vi) identified by a member of prison staff or via self-report as having recently been involved in an actual or expressed episode of verbal or physical harm to others or objects or as at risk of engaging in violent behavior due to violence ideation. Eligibility was confirmed from self-reported responses to the following four questions: (1) Over the past 3 months have you thought about killing yourself? (2) Over the past 3 months have you tried to kill yourself? (3) Over the past 3 months have you thought about hurting somebody else? (4) Over the past 3 months have you tried to hurt somebody else? A positive response to any of these four questions confirmed eligibility for the study. Prisoners were excluded where they had been assessed by the prison's security department and deemed at too high-risk due to security intelligence to move around the prison estate and mix with other prisoners, or to be seen by the researcher without special security measures, including those residing in segregation units. Participants received no payment or privileges for participating which was entirely voluntary. Participants did not lose any usual payments (e.g., prison wages) by participating in this study.

An a-priori power calculation was conducted using Stata to estimate the required number of participants to achieve an effect size of 0.3 in exploring the core hypothesis of this study (H1). An effect size of 0.3 was chosen based on previous research on the bivariate relationship between alexithymia and suicide ideation (14) and would be considered to have clinical utility. The input parameters used for the calculation were: effect size of 0.3, probability level of 0.05 and 80% power. This analysis indicated that a minimum sample of 80 was required.

### Measures

The following measures were administered as part of a larger battery of questionnaires.

#### Alexithymia

The Toronto Alexithymia Scale (TAS-20) (13) is a 20 item self-report measure of alexithymia comprised of three main factors: Difficulty Identifying Feelings (DIF), Difficulty Describing Feelings (DDF) and Externally Oriented Thinking. Example items include: “*I have feelings that I can't quite identify”* (DIF), “*It is difficult for me to find the right words for my feelings”* (DDF) and “*I prefer talking to people about their daily activities rather than their feelings”* (EOT). Respondents rate their agreement using a five-point likert scale ranging from 1 (strongly disagree) to 5 (strongly agree), with higher scores indicating greater difficulties with alexithymia. The alpha coefficient for the current sample was 0.81. The alpha coefficient for the DIF subscale was 0.77, for the DDF subscale was 0.75 and for the EOT subscale was 0.42. These coefficients are in line with the authors' analyses (13), apart from the EOT subscale which has a lower alpha coefficient in the current sample. Despite this, reviews have shown that it is common in the literature for studies to report a lower alpha coefficient for the EOT subscale (55). Pertinent to this sample, and subsequent to the conduct of the current study, the EOT subscale has recently been found to have an unacceptable alpha coefficient within an offender population (56).

#### Suicide Ideation

The Adult Suicide Ideation Questionnaire (ASIQ) (57) is a 25 item self-report measure of suicide ideation and behavior in adults. Example items include “*I thought that if I had a chance, I would kill myself”*. Respondents rate the frequency of suicidal thoughts or behavior during the past month using a 7-point Likert scale for each item ranging from 0 (never had the thought) to 6 (almost every day), with higher scores indicating a greater frequency of suicidal thoughts. The alpha coefficient for the current sample was 0.96, in line with the authors' analyses of internal consistency in male psychiatric outpatients (57).

#### Suicide Behavior

Suicide behavior was measured using a bespoke 6 item questionnaire. The items used to measure suicide behavior in the current paper were: “*Over the past 2 weeks, how many times have you attempted to cause deliberate harm to yourself?”* and “*Over the past 2 weeks, how many times have you made an attempt to kill yourself in which you had at least some intent to die?”*. Due to a high frequency of nil responses, suicide behavior was treated as a dichotomous variable; those who had zero suicide behaviors in the past 2 weeks, and those who had one or more suicide behaviors.

#### Violence Ideation

The Schedule of Imagined Violence (SIV) (58) is an 8 item self-report measure, which aims to provide descriptive data on an individual's thoughts about violence. Example items include “*Do you ever have daydreams or thoughts about physically hurting or injuring some other persons?”*. In the current study, a scoring system was devised (Appendix A) enabling a total score of 0–19, such that a higher score in SIV reflected respondents experiencing violent thoughts more recently, at a greater frequency and for a longer chronicity. Since there were two peaks in the distribution of SIV scores, this suggested two distinct categories of responders. Therefore, SIV scores were categorized according to a median split, so that those who score up to 9 were classified as “no/low violence ideation” and those who score 10 and above were classified as “high violence ideation.”

#### Violence Behavior

Violence behavior was measured using a bespoke 5-item questionnaire. The items used to measure violence behavior in the current paper were: “*Over the past 2 weeks, how many times have you threatened to hurt somebody other than yourself?” “Over the past 2 weeks, how many times have you been verbally aggressive to somebody other than yourself?” “Over the past 2 weeks how many times have you been aggressive with inanimate objects?” “Over the past 2 weeks, how many times have you been violent towards somebody other than yourself?”*. Due to a high frequency of nil responses, violence behavior was treated as a dichotomous variable; those who had zero violent behaviors in the past 2 weeks, and those who had one or more violent behaviors in the past 2 weeks.

#### Dual Harm Ideation

Respondents were categorized into three groups; those who had experienced suicide ideation alone in the past month (suicide ideation, *N* = 25), those who had experienced violence ideation alone in the past 2 months (violence ideation, *N* = 13) and those who had experienced both (dual harm ideation, *N* = 29). In accord with recommendations stipulated in the Adult Suicidal Ideation Questionnaire manual (57), suicide ideation scores up to 31 were considered to indicate mild-moderate suicide ideation and scores of 31 and above were considered to indicate severe suicide ideation. Respondents' violence ideation scores were divided according to a median split, such that those who scored up to 9 were considered to experienced mild-moderate violence ideation, and those who scored 10 or above were considered to experience severe violence ideation. Respondents who experienced severe ideation for both suicide and violence were identified as having experienced “dual harm” ideation.

#### Dual Harm Behavior

Respondents were categorized into three groups; those who had experienced suicide behaviors alone (*N* = 4), those who had experienced violent behaviors alone (*N* = 25) and those who had experienced both behaviors, i.e. “dual harm” (*N* = 12).

#### Depression

The Beck Depression Inventory (BDI-II) (59) is a 21-item self-report scale of depressive symptoms within the past 2 weeks. Example topics covered include sadness, pessimism and loss of interest. Responses are weighted with a score between 0 and 3 based on emotional content of the response, 0 indicating a bright mood or lack of depressive symptoms and 3 indicating a highly depressive reaction. Thus, higher scores indicate greater depression symptoms. The alpha coefficient for the current sample was 0.90, this is in line with the coefficients reported by the authors of the scale (60).

#### Hopelessness

The Beck Hopelessness Scale (BHS) (61) is a 20-item self-report measure of hopelessness within the past 2 weeks. Example items include “*My future seems dark to me.”* Respondents are asked to respond true or false, with higher scores indicating greater thoughts and feelings of hopelessness. The alpha coefficient for the current sample was 0.93, which is similar to that reported by the scale authors (61).

#### Anger

The Novaco Anger Scale (NAS) (62) is a 60-item self-report scale focussing on how an individual experiences anger. There are four subscales; cognitive, arousal, behavioral and anger regulation. Example items include: “*Once something makes me angry, I keep thinking about it”* (cognitive), “*Some people would say that I'm a hothead”* (arousal), “*When I get mad, I can easily hit someone”* (behavioral) and “*If I feel myself getting angry, I can calm myself down”* (anger regulation). Respondents are asked to respond using a Likert scale of 1 (never true), 2 (sometimes true) or 3 (always true). Higher scores indicate higher levels of anger. The alpha coefficient for the current sample was 0.91, this is similar to the alpha coefficient reported for hospitalized inpatients in the authors' analyses (62).

#### Impulsivity

The Dickman Impulsivity Inventory (DII) (63) is a 23-item self-report measure of impulsivity, with two subscales: functional and dysfunctional impulsivity. Example items include: “*People have admired me because I can think quickly.”* (functional) and “*I often get into trouble because I don't think before I act”* (dysfunctional). Respondents are asked to state whether statements are true or false for them. Only the dysfunctional subscale is used in the current study. The alpha coefficient for the dysfunctional subscale with the current sample was 0.78, which is similar to that reported by the authors of the scale (63).

### Procedure

Potential participants met with the researcher and were given an opportunity to consider the participant information sheet, typically no <24 h before signing a consent form. Questionnaires were completed in a private room in the prison. To overcome issues of poor literacy, questionnaires were read aloud to all participants and responses were recorded by the researcher.

### Ethics

The authors assert that all procedures contributing to this work comply with the ethical standards of the relevant national and institutional committees on human experimentation and with the Helsinki Declaration of 1975, as revised in 2008. All procedures involving human subjects were approved by the East of Scotland Research Ethics Service (18/ES/0022) which specializes in research involving prisoner participants. Written informed consent was obtained from all participants.

### Statistical Methods

Data were analyzed using IBM SPSS statistics, version 25. Initially, the data were inspected for errors and missing items. Respondents who had more than 20% of questionnaire items missing were excluded from analysis of that scale. Scores were prorated for respondents with <20% of items missing. Individual item scores were prorated by calculating an average item score for that respondent on either the full scale, or where possible, on the subscale (13, 62).

Normality, linearity and homoscedasticity were assessed graphically. Where residuals were considered to violate the assumption of normality, bootstrapping was performed with 1,000 iterations. Data were considered to violate the assumption of multicollinearity where correlations exceeded 0.7(64). In addition, multicollinearity was assessed by calculating a tolerance and VIF value. Tolerance values of <0.10 and VIF values of above 10 were considered to violate the assumption of multicollinearity (64).

In relation to hypothesis 1, bivariate correlations were conducted to determine the relationship between alexithymia, and subscales, with suicide ideation. Following this, two standard multiple regressions were conducted. Model 1a included the independent variable of alexithymia total score, confounders of depression, hopelessness and anger, and the dependent variable of suicide ideation. Model 1b used alexithymia subscales instead of alexithymia total score.

In relation to hypothesis 2, two multivariate binary logistic regression models were conducted. Model 2a included the independent variable of alexithymia total score, confounders of impulsivity and anger, and the dependent variable of violence ideation. Model 2b used alexithymia subscales instead of alexithymia total score.

In relation to hypothesis 3, two multivariate binary logistic regression models were conducted. Model 3a included alexithymia total score as the independent variable, anger as a confounder, and violence ideation as a dependent variable. Model 3b substituted alexithymia total score for alexithymia subscales.

In relation to hypothesis 4, two multivariate binary logistic regression models were conducted. Model 4a included alexithymia total score as the independent variable, anger as a confounder, and violent behavior as the dependent variable. Model 4b used alexithymia subscales instead of alexithymia total score.

In relation to hypothesis 5, an ANOVA was used to determine significant differences in score for alexithymia, including subscales, depression, hopelessness and anger between those who experienced suicide or violence ideation alone and dual harm ideation. A *post-hoc* Tukey test was used to explore comparisons.

In relation to hypothesis 6, an ANOVA was used to determine significant differences in alexithymia and subscales, impulsivity and anger between those who experienced suicide or violence behaviors only and dual harm behaviors. A *post-hoc* Tukey test was used to explore comparisons.

## Results

### Participant Characteristics

One-hundred and fifty-three prisoners were identified as potentially eligible for participation in the study. Of these, 110 were examined for eligibility. Reasons for not being examined for eligibility included: potential participant not interested in participating (*n* = 17), not being able to make contact with the potential participant (*n* = 15), recruitment ceasing before assessing for eligibility (*n* = 10) and staff advising not to see potential participant due to risk (*n* = 1). Unfortunately, it was not possible to collect information about individuals who refused to take part, due to refusals occurring at different stages. For instance, some refusals occurred before being approached by the researcher, and therefore ethical approvals did not allow for the collection of data from these individuals. Of those examined for eligibility, 100 were confirmed as eligible for participation. The final sample comprised 80 participants all of whom were included within the analyses. Reasons for not participating in assessments included: individual not wanting to participate (*n* = 13), being transferred out of the prison (*n* = 5), researcher not being able to make contact with participant (*n* = 1) and staff preventing researcher from accessing participant due to risk (*n* = 1).

Participants were predominantly White British (89%) male prisoners aged between 21 and 56 years old (Median = 32, range = 21–56). The median age at which participants had first been imprisoned was 18 years old (range = 12–49), and participants had been incarcerated on an average of 4 times (range 1–30). According to the UK Home Office Crime Types (65), the most frequent offenses as recorded in prison records were: violent offenses (*N* = 56); acquisitive offenses (*N* = 10); drug offenses (*N* = 7); vandalism and criminal damage (*N*=3) and fraud and forgery (*N* = 1). According to medical records, a total of 37.5% of participants had at least one psychiatric diagnosis, including personality disorder (*N* = 9); depression (*N* = 7); ADHD (*N* = 3); schizophrenia/psychosis (*N* = 3); PTSD (*N* = 1) with the remaining participants either missing data (*N* = 15) or experiencing a comorbidity of diagnoses (*N* = 7). Medical records indicate that 50% of participants were taking medication for their mental health at the time of participation. The majority of participants were prescribed antidepressants (80%), whilst 25% were prescribed antipsychotics^2^. Participants reported a median number of lifetime custodial suicide attempts as 2 (range 0–40) and lifetime custodial violent incidents as 4 (range 0–400).

### Descriptive Statistics

The median scores, interquartile ranges and correlation coefficients from the questionnaire measures are presented in Table 1. The number of participants providing scores for each measure ranged from 77 to 80, except for hopelessness where only 60 participants completed the measure. This is due to this measure being subsequently added to the assessment battery after recruitment had begun.

**Table 1:** Mean scores, standard deviations, range, sample size, and correlation coefficients for key variables.

	**Median**	**IQR(25–75%)**	**Range**	**N**	**1**	**2**	**2a**	**2b**	**2c**	**3**	**4**	**5**
**1. Suicide ideation**	50.0	24.0–75.0	4–149	79	-	0.39*^b^	0.38*^b^	0.37*^b^	0.12 ^b^	0.68*^b^	0.57*^b^	0.39*^b^
**2. Alexithymia**	65.5	53.3–74.0	26–90	80		-	0.85*^a^	0.81*^b^	0.70*^a^	0.55*^a^	0.59*^b^	0.55*^a^
2a. Difficulty identifying feelings	24.0	18.0–28.0	7–35	80			-	0.57*^b^	0.32*^a^	0.54*^a^	0.52*^b^	0.45*^a^
2b. Difficulty describing feelings	19.0	14.0–23.0	5–25	80				-	0.40*^b^	0.42*^b^	0.44*^b^	0.56*^b^
2c. Externally oriented thinking	22.5	19.0–26.8	8–34	80					-	0.27 ^a^	0.40*^b^	0.32*^a^
**3. Depression**	33.0	20.5–42.0	3–59	77						-	0^.^65*^b^	0.39*^a^
**4. Hopelessness**	10.0	5.0–14.8	0–20	60							-	0.30 ^b^
**5. Anger**	106.0	89.0–118.0	62–140	77								-

### Suicide Outcomes

#### Suicide Ideation

Suicide ideation scores were significantly correlated with scores for alexithymia (*r*^3^ = 0.39, *N* = 79, *p* < 0.001, difficulty identifying feelings (*rs* = 0.38, *N* = 79, *p* = 0. 001), difficulty describing feelings (*rs*^4^ = 0.37, *N* = 79, *p* = 0.001), depression (*r* = 0.68, *N* = 76, *p* = 0.000), hopelessness (*r* = 0.57, *N* = 60, *p* < 0.001) and anger (*r* = 0.39, *N* = 76, *p* < 0.001). Externally oriented thinking was not significantly correlated with suicide ideation (*r* = 0.12, *N* = 79, *p* = 0.277).

Multiple regression models were used to examine the contributions of alexithymia, alexithymia subscales, depression, hopelessness and anger in the prediction of suicide ideation severity (Table 2). Preliminary analyses confirmed that there were no violations of the assumptions of normality, linearity, multicollinearity, and homoscedasticity. Model 1a includes alexithymia total score, depression, hopelessness and anger. The total variance explained by the model as a whole was 51% [*F*_(4,55)_ = 14.19, *p* < 0.001, *R*^2^ = 0.51]. In model 1a, only depression (*B* = 1.38, 95%CI 0.81–1.92, *p* = 0.001) was a significant predictor of suicide ideation. In model 1b, the total alexithymia score was replaced by the subscales of alexithymia. The total variance explained by this model as a whole was 53% [*F*_(6,53)_ = 9.75, *p* < 0.001, *R*^2^ = 0.53]. Similar to model 1a, only depression was found to be a significant predictor of suicide ideation [*B* = 1.38, 95%CI 0.76–1.90, *p* = 0.001]. The results of the regression analyses therefore reject H1.

**Table 2:** Suicide ideation hierarchical multiple regression (*N* = 60).

**Step**	**Predictor variables**	**Unstandardized B**	**95% CI^a^**	**Standard error**	**Standardized B**	**T**	**p**
**1a**	Alexithymia	−0.27	−0.96–0.39	0.35	−0.12	−0.89	0.447
	Depression^*^	1.38	0.81–1.92	0.27	0.54	4.05	0.001
	Hopelessness	1.11	−0.17–2.62	0.71	0.20	1.48	0.134
	Anger	0.31	−0.18–0.77	0.25	0.18	1.58	0.255
**1b**	Alexithymia – difficulty identifying feelings	−0.38	−1.67–1.05	0.70	−0.08	−0.63	0.617
	Alexithymia – difficulty describing feelings	0.64	−1.11–2.47	0.90	0.10	0.76	0.477
	Alexithymia – externally oriented thinking	−1.01	−2.31–0.26	0.63	−0.16	−1.45	0.114
	Depression^*^	1.38	0.76–1.90	0.29	0.54	3.95	0.001
	Hopelessness	1.11	−0.25–2.71	0.76	0.20	1.48	0.145
	Anger	0.26	−0.24–0.68	0.24	0.15	1.27	0.289

a *Bootstrapped confidence intervals*.

* *p < 0.001*.

#### Suicide Behavior

There were 43 participants who had not experienced any suicide behaviors in the past 2 weeks, nineteen participants with data missing and eighteen participants who had experienced at least one suicide behavior in the past 2 weeks. Multivariate binary logistic regression was conducted to assess the impact of alexithymia and subscales, impulsivity and anger on suicide behavior. In model 2a, alexithymia total score, impulsivity and anger were entered into the model. The full model containing all predictors was statistically significant x^2^ (3, *N* = 60) = 10.88, *p* = 0.012, indicating that the model was able to distinguish respondents who reported suicidal behaviors in the past 2 weeks from those who did not. The model as a whole explained between 16.6% (Cox & Snell R Square) and 23.5% (Nagelkerke R square) of the variance in suicide behaviors, and correctly classified 75% of cases. Only alexithymia total score was a significant contributor to the model. For every unit increase in alexithymia total score, the odds of having experienced suicidal behavior in the past 2 weeks increased by 8% (OR = 1.08, 95% CI = 1.01–1.15, *p* = 0.020. In model 2b, which included alexithymia subscales instead of alexithymia total score, only difficulty identifying feelings was a significant contributor to the model, with a 15% increased odds of suicidal behavior for every unit increase in difficulty identifying feelings (OR = 1.15, 95%CI = 1.02–1.29, *p* = 0.024). These regression models therefore lend support to H2.

### Violence Outcomes

#### Violence Ideation

The median SIV score for violence ideation was 10.0 (IQR = 2.0–16.0, *N* = 79). The number of participants categorized as experiencing “no/low” violence ideation was *N* = 37 and *N* = 42 for “high violence ideation.”

Multivariate binary logistic regression was used to assess the impact of alexithymia, and subscales, and anger on the odds of experiencing a “high level” of violence ideation. Model 3a, which contained alexithymia total score and anger, was found to be statistically significant x^2^ (2, *N* = 77) = 14.88, *p* < 0.001, indicating that the model was able to distinguish between those experiencing low/no violence ideation and those experiencing “high” violence ideation. However, the only significant contributor in this model was anger (OR = 1.06, 95% CI = 1.02–1.10, *p* = 0.002). In model 3b, which contained alexithymia subscales and anger, anger was again the only predictor to be significantly associated with violence ideation (OR = 1.07, 95% CI = 1.03–1.11, *p* < 0.001). These models therefore reject H3.

#### Violence Behaviors

There were 24 participants who had not experienced any violent behaviors in the past 2 weeks, nineteen participants who had data missing and 37 participants who had experienced at least one violent behavior in the past 2 weeks.

In model 4a, multivariate binary logistic regression was used to assess the impact of alexithymia, impulsivity and anger on the likelihood that respondents had experienced violent behavior in the past 2 weeks. Whilst the model itself was statistically significant (x^2^ (3, *N* = 60) = 8.74, *p* = 0.033) no significant odds ratios were found for any predictors. The same was true of model 4b which contained alexithymia subscales, anger and impulsivity. H4 was therefore rejected.

### Dual Harm

#### Dual Harm Ideation

An ANOVA was conducted (Table 3) to examine the differences between those experiencing severe suicide ideation alone in the past month (*N* = 25), those experiencing severe violence ideation alone in the past 2 months (*N* = 13) and those experiencing “dual harm” ideation, i.e., severe suicide *and* violence ideation (*N* = 29). There were no significant differences observed in alexithymia or any subscales across the three groups, therefore rejecting H5. For both depression and hopelessness scores, those who had experienced suicide ideation alone or dual harm ideation had higher scores than those who had experienced violence ideation alone (Depression (*F* = 14.13, *p* < 0.001): suicide ideation *M* = 37.2, dual harm ideation *M* = 36.6, violence ideation *M* = 19.6. Hopelessness (*H* = 8.50, *p* = 0.014): suicide ideation *M* = 12.5, dual harm ideation M=10.9, violence ideation *M* = 5.5).

**Table 3:** ANOVA of dual harm ideation.

**Predictor**	**F/H value**	**p**	**Suicide ideation (*N* = 25)**	**Violence ideation (*N* = 13)**	**Dual harm ideation (*N* = 29)**	**Sig difference between**
**1. Alexithymia**	1.79 ^a^	0.175	63.0 (13.3)	60.6 (13.9)	68.1 (13.0)	–
1a. Difficulty identifying feelings	1.24 ^a^	0.297	23.3 (7.1)	22.2 (5.0)	25.3 (6.8)	–
1b. Difficulty describing feelings	4.09 ^b^	0.129	18.0 (5.2)	16.4 (4.7)	19.5 (4.9)	–
1c. Externally oriented thinking	0.72 ^a^	0.491	21.6 (4.6)	22.1 (6.1)	23.3 (5.1)	–
**2. Depression**	14.13 ^a^	<0.001	37.2 (11.8)	19.6 (11.3)	36.6 (8.7)	Suicide > violence. Dual harm > violence.
**3. Hopelessness**	8.50 ^b^	0.014	12.5 (5.9)	5.5 (5.9)	10.9 (5.4)	Suicide > violence. Dual harm > violence.
**4. Anger**	2.96 ^a^	0.059	102.4 (17.0)	107.8 (18.8)	113.5 (15.2)	–

a *parametric*.

b *non-parametric*.

#### Dual Harm Behavior

An ANOVA was conducted (Table 4) to examine the differences between those experiencing only suicide behaviors in the past 2 weeks (*N* = 4), those experiencing only violent behaviors in the past 2 weeks (*N* = 25) and those experiencing suicide *and* violent behaviors or “dual harm” behaviors (*N* = 12). There were no significant differences between the three groups on any of the following variables; alexithymia, difficulty identifying feelings, difficulty describing feelings, externally oriented thinking, impulsivity or anger. H6 was therefore rejected.

**Table 4:** ANOVA of dual harm behavior.

**Predictor**	**F/H value**	**p**	**Suicide (*N* = 4)**	**Violence (*N* = 25)**	**Dual harm (*N* = 12)**	**Sig difference between**
**1. Alexithymia**	0.53 ^a^	0.592	70.7 (19.2)	64.8 (10.2)	68.7 (18.3)	–
1a. Difficulty identifying feelings	2.72 ^a^	0.078	24.2 (10.4)	22.5 (5.5)	27.8 (7.1)	–
1b. Difficulty describing feelings	0.43 ^b^	0.807	20.5 (5.1)	19.1 (4.3)	18.1 (6.5)	–
1c. Externally oriented thinking	0.55 ^a^	0.580	26.0 (5.9)	23.2 (4.5)	22.8 (6.7)	–
**Impulsivity**	2.18 ^b^	0.337	7.2 (5.1)	9.6 (1.6)	7.2 (4.2)	–
**Anger**	0.23 ^a^	0.793	102.0 (21.4)	108.5 (17.6)	107.6 (16.8)	–

a *parametric*.

b *non-parametric*.

## Discussion

### Summary of Findings

This study found that alexithymia was a univariate predictor of suicide ideation, although no longer significant following the inclusion of other well-known correlates of suicide ideation in a multivariate model. Alexithymia was found to be a significant multivariate predictor of suicide behavior; for every unit increase in alexithymia total score, the odds of having experienced suicidal behavior in the past 2 weeks increased by eight percent. Neither alexithymia, nor subcomponents, were found to be significant multivariate predictors of violence ideation or behavior. There were no differences in alexithymia or subcomponents between those that experienced suicidal/violent ideation only and those who experienced both.

### Comparisons With Wider Literature

Hypothesis one of this study was rejected. Although a significant bivariate correlation was found between alexithymia and suicide ideation, in regression models which accounted for known confounders of this relationship such as depression, hopelessness and anger, neither alexithymia nor its subscales were significantly associated with suicide ideation. Within the correlation matrix, the subcomponents of “difficulty identifying feelings” and “difficulty describing feelings” were found to be significantly associated with suicide ideation, though the subcomponent of “externally oriented thinking” was not found to be significantly associated. This reflects previous meta-correlation research which has found a significant association between alexithymia total scores, difficulty identifying feelings and difficulty describing feelings with suicide ideation (14). Previous research on the multivariate relationship between alexithymia and suicide ideation is mixed (14), with some finding a relationship remained when accounting for depression (66) and others finding no relationship (67). The findings of this study suggest that there is no relationship between alexithymia or subcomponents and suicide ideation in male prisoners when accounting for known correlates of depression, hopelessness, and anger.

Hypothesis two in the current study was accepted; both alexithymia total score and the subscale of difficulty identifying feelings were found to be associated with suicide behavior, when controlling for confounders of impulsivity and anger. This finding is supported by previous meta-analyses which have found a small to medium effect size for the relationship between alexithymia and suicide behavior (14–16). These meta-analyses have found associations to be strongest with subcomponents “difficulty identifying and describing feelings,” which is partially supported by the current findings which found the strongest association with “difficulty identifying feelings.”

Hypothesis three in this study was rejected. Neither alexithymia total score nor any subscales were found to be associated with violence ideation. Hypothesis four in this study was also rejected; neither alexithymia total score nor alexithymia subscales were found to predict an increased odds of having experienced violent behavior in the past 2 weeks. Previous research has tended to focus on the relationship between alexithymia and “anger” or “aggression” and has not distinguished between violence ideation and behaviors as the present study has done. Such a body of research, however, contrasts with the current findings in that a significant relationship has been found between alexithymia and aggression in a range of populations (22–24, 27, 68, 69). There have been relatively few studies which explore the multivariate relationship between alexithymia and violence, however one study, contrary to the findings in the present paper, found that the relationship between “difficulty identifying feelings” and aggression remained when accounting for both anxiety and depression (22).This research does not consider the covariates of anger and impulsivity as the present study has done, and this may therefore explain why alexithymia was not found to be associated with violence in the present study.

Hypotheses five and six in this study were rejected; those who experienced dual harm ideation/behavior were not found to have higher scores in alexithymia (or subcomponents) than those who experienced suicidal or violent ideation/behavior alone. To date, there has been no research which directly investigates the relationship between alexithymia and dual harm. Despite this, previous research has found, in contrast to the present findings, that individuals exhibiting self-regulation difficulties are more likely to experience dual harm than self-harm alone (70). These differences in findings may be due to methodological limitations of the present study in relation to small sample size.

It is worth considering as a whole the lack of positive findings reported in this study. Although this study has been unable to reject the null hypothesis, this does not mean that the null hypothesis is true (i.e., that there is *not* a relationship between alexithymia and suicide, violence and dual harm). For instance, it may be that a mediational or interactional relationship exists between alexithymia, confounders and dependent variables. Unfortunately, the present study was unable to explore these analyses due to limited sample sizes. The fact that this study reports results that contrast with those in the extant literature further suggests that there may be a more complicated relationship between alexithymia, confounders, suicide and violence, that the present study has been unable to elucidate.

Alternatively, it may be prudent to conclude, based on the findings of this study, that alexithymia should not be viewed as a promising predictor of suicide or violence in male prisoners. Such a finding is supported by previous research which was ambivalent regarding the nature of confounding variables in the relationship between alexithymia and suicide ideation (14). Moreover, the fact that there as many as 23 psychosocial risk factors implicated in both suicide and violence (32, 33) suggests that there may be alternative risk factors which are more relevant in predicting suicide and violence amongst prisoners. The findings presented here suggest that depression is a strong risk factor for suicide, whilst anger is a strong risk factor for violence.

### Strengths, Limitations, and Areas for Future Research

The present study is the first to explore the relationship between alexithymia, suicide, violence and dual harm amongst male prisoners. The inclusion of both suicide and violence outcomes in this study is a strength, as previous research has highlighted that those who harm both themselves and others may represent a distinct group of individuals, particularly amongst male prisoners (42, 71). Despite this, there is little research which considers risk factors for both outcomes. Furthermore, the separation of outcomes into “ideation” and “behaviours” is a strength of this study, given that previous research has suggested risk factors may differ and have therefore called for research to focus on an “ideation to action” framework (72, 73). This study utilized a patient and public involvement group which was perceived to enhance the quality of the study in several ways including an enhanced recruitment rate and alternative interpretations of the results, and also ensure that findings are disseminated to participants in a manner that is easy to understand. The reporting of this patient and public involvement is also a strength of the current study, given the lack of detail that is often provided in academic journals. In this study, 80% of those that were eligible to participate agreed to take part in the study. This is encouraging and suggests that research exploring suicide and violence amongst male prisoners in this manner appears feasible and acceptable. Finally, the quality of the data in this study is a strength. Data were collected via verbal interview, which although time consuming, has led to a greater accuracy of data as well as small amounts of missing data.

Despite this, there are some limitations to consider in the present study, which should be recognized when interpreting the findings. First, this study used a cross-sectional design and it is therefore difficult to determine the direction of the relationship between predictor and outcomes. Indeed, there is research to suggest that alexithymia may be “secondary” and form as a *result* of, rather than a cause of, psychological distress (74–76). It is therefore plausible that alexithymia may be experienced as a result of suicide and/or violence, as opposed to a preceding experience. Future research should therefore use longitudinal or micro-longitudinal methods (such as experience sampling methodology) to determine the direction of the relationship between alexithymia, suicide, violence, and dual harm.

A second limitation of this study is its relatively small sample size. This is particularly problematic given the small number of people that reported to have engaged in suicidal or violent behaviors. It is worth noting that hypotheses two to six in this study were not guided by an a-priori power calculation, due to their exploratory nature, and this might therefore lend them to greater probability of type 2 error. Moreover, the small sample size meant that it was not possible to conduct complex analyses such as interaction effects between predictors, due to these analyses being underpowered and subject to type 2 error. Future research should therefore aim to recruit sufficiently larger samples to explore these research questions in greater detail, including examining the interaction effects between alexithymia and known predictors of suicide and violence.

Related to the small sample size, a third limitation of this study is its lack of generalizability. The sample in the present study was predominantly White British (89%) which is in disproportion to reports which show that around 73% of male prisoners in England and Wales are White British (77). Furthermore, the present study excluded people residing in segregation units which may have biased the sample. Additionally, a large proportion of participants in this study were incarcerated for non-violent offenses, which may therefore represent only a subset of male prisoners more broadly. This is particularly pertinent given the focus of this study on violence, and may therefore have skewed the present findings. Future research should therefore aim to recruit a more representative sample of male prisoners to explore these research questions. Moreover, it may be of relevance to explore similar research questions in different, but related, populations such as young offenders and female prisoners.

Finally, the data collected in the present study were based on self-report, which may have led to response bias or distortions (78). Despite this, it has been argued that self-report data allows for a complete and non-judgemental assessment (79) and moreover the accuracy of record data is often disputed (80).

### Clinical Implications

Given the lack of positive findings in this study, it is difficult to provide clinical implications with confidence. Therefore, as already suggested, the main priority should be to conduct future research into the role of alexithymia, and confounders, upon suicide and violence outcomes. Despite this, the current study found that alexithymia was significantly associated with suicidal behavior, even when accounting for impulsivity and anger. These findings therefore suggest that there is potential utility in screening individuals for alexithymia who may already score highly on other risk factors for suicide including depression, hopelessness, anger and impulsivity. This may help to identify those who are at an elevated risk of suicide. Screening should be conducted using established measures of alexithymia such as the Toronto Alexithymia Scale (13).

Furthermore, individuals who score above the threshold for alexithymia should be given tailored intervention to help them to identify and discuss their feelings. Previous research has found that an alexithymia specific treatment comprised of mindfulness and metallization techniques led to a decrease in alexithymia scores with sex offenders (81). The findings of the current study suggest that such a targeted intervention may also be directly useful in reducing rates of suicide amongst male prisoners. Furthermore, it is well-reported that individuals with alexithymia struggle to engage in psychological therapies (82–85). Providing tailored support to assist with difficulties in identifying and describing feelings may therefore render individuals more able to engage in well-established psychological talking therapies for suicide and violence prevention, for instance cognitive-behavioral suicide prevention which has previously been found effective amongst a male prisoner population (86).

## Data Availability Statement

The datasets presented in this article are not readily available because of issues of confidentiality and anonymity. Requests to access the datasets should be directed to daniel.pratt@manchester.ac.uk.

## Ethics Statement

The studies involving human participants were reviewed and approved by East of Scotland Research Ethics Services. The patients/participants provided their written informed consent to participate in this study. Written informed consent was obtained from the individual(s) for the publication of any potentially identifiable images or data included in this article.

## Author Contributions

LH was responsible for data collection, wrote a first draft of the manuscript with all authors contributing to the final manuscript. LH, L-AC, and DP led on data analysis. All authors contributed to the formulation of research questions and designing the study.

## Conflict of Interest

The authors declare that the research was conducted in the absence of any commercial or financial relationships that could be construed as a potential conflict of interest.

## Footnotes

^1^All UK prisons have a safer custody team, responsible for the management of prisoners at risk of harm to self, to others and from others.

^2^Does not total 100% due to participants being prescribed more than one type of medication.

^3^r = Pearson's correlation.

^4^rs = Spearman's correlation.
