# Supplementary material for: A Cross-Sectional Study Investigating the Relationship Between Alexithymia and Suicide, Violence, and Dual Harm in Male Prisoners
Source: Front Psychiatry. 2021 Apr 29;12:670863. doi: 10.3389/fpsyt.2021.670863 (PMC8116549; doi:10.3389/fpsyt.2021.670863)
Supplement: Supplementary file 1 [file Data_Sheet_1.docx]

**Appendix A – Schedule of Imagined Violence scoring scale**

1. Do you ever have daydreams or thoughts about physically hurting or injuring some other persons?

| No | 0 |
| --- | --- |
| Yes | 1 |

1. When was the last time you had such a daydream or thought?

| Don’t know | 0 |
| --- | --- |
| Longer than two months ago | 1 |
| In the past two months | 2 |
| In the past month | 3 |
| In the past three to seven days | 4 |
| In the past two days | 5 |
| Today | 6 |

1. How often have you had these daydreams or thoughts in the past two months?

| Don’t know | 0 |
| --- | --- |
| About once in the past two months | 1 |
| Two or three times in the past month | 2 |
| A few times a month | 3 |
| Once a week | 4 |
| Several times a week | 5 |
| Once a day | 6 |
| Several times a day | 7 |

1. When did you start having these daydreams or thoughts?

| Don’t know | 0 |
| --- | --- |
| During the past month | 1 |
| During the past 1-3 months | 2 |
| During the past 3-6 months | 3 |
| During the past 6-12 months | 4 |
| Before the past year | 5 |

Grisso T, Davis J, Vesselinov R, Appelbaum PS, Monahan J. Violent thoughts and violent behavior following hospitalization for mental disorder. J Consult Clin Psychol. 2000;68(3):388.
